# Cyclic cidofovir (cHPMPC) prevents congenital cytomegalovirus infection in a guinea pig model

**DOI:** 10.1186/1743-422X-3-9

**Published:** 2006-03-01

**Authors:** Mark R Schleiss, Jodi L Anderson, Alistair McGregor

**Affiliations:** 1 Division of Infectious Diseases, University of Minnesota Department of Pediatrics, Center for Infectious Diseases and Microbiology Translational Research, 2001 6^th ^Street SE, McGuire Translational Research Facility, Minneapolis, Minnesota 55455, USA

## Abstract

**Background:**

Congenital cytomegalovirus (CMV) infection is a major public health problem. Antiviral therapies administered during pregnancy might prevent vertical CMV transmission and disease in newborns, but these agents have not been evaluated in clinical trials. The guinea pig model of congenital CMV infection was therefore used to test the hypothesis that antiviral therapy, using the agent agent cyclic cidofovir (cHPMPC), could prevent congenital CMV infection.

**Results:**

Pregnant outbred Hartley guinea pigs were challenged in the early-third trimester with guinea pig CMV (GPCMV) and treated with placebo, or the antiviral agent, cyclic cidofovir. To optimize detection of vertical infection, an enhanced green fluorescent protein (eGFP)-tagged virus was employed. Compared to placebo, cyclic cidofovir-treated dams and pups had reduced mortality following GPCMV challenge. The magnitude of GPCMV-induced maternal and fetal mortality in this study was reduced from 5/25 animals in the placebo group to 0/21 animals in the treatment group (p = 0.05, Fisher's exact test). By viral culture assay, antiviral therapy was found to completely prevent GPCMV transmission to the fetus. In control pups, 5/19 (26%) were culture-positive for GPCMV, compared to 0/16 of pups in the cyclic cidofovir treatment group (p < 0.05, Fisher's exact test).

**Conclusion:**

Antiviral therapy with cyclic cidofovir improves pregnancy outcomes in guinea pigs, and eliminates congenital CMV infection, following viral challenge in the third trimester. This study also demonstrated that an eGFP-tagged recombinant virus, with the reporter gene inserted into a dispensable region of the viral genome, retained virulence, including the potential for congenital transmission, facilitating tissue culture-based detection of congenital infection. These observations provide support for clinical trials of antivirals for reduction of congenital CMV infection.

## Background

Congenital cytomegalovirus (CMV) infection is a major public health problem. Transmission of CMV *in utero *results in substantial long-term morbidity in newborns, including mental retardation and sensorineural hearing loss (SNHL; reviewed in [1]). Treatment of the affected newborn with the anti-CMV nucleoside analogue, ganciclovir, improves the outcome of SNHL, but the response is incomplete, and significant sequelae may persist even following completion of antiviral therapy [2]. These observations provide support for studying the approach of administering antiviral agents administered prior to delivery, with the goal of preventing acquisition of infection *in utero*. Such therapy could potentially be employed in pregnant women in the setting of documented fetal CMV infection, as demonstrated by seroconversion to CMV, or by amniotic fluid analysis confirming the presence of CMV genome. Although this intervention has been attempted and described in a number of case reports [3-5], it is not clear whether *in utero *therapy for CMV is effective in interrupting vertical transmission, or reducing disease.

Ideally, antiviral therapy strategies designed to prevent congenital viral transmission would be evaluated in animal models prior to human clinical trials. One attractive model is the guinea pig cytomegalovirus (GPCMV) model [reviewed in [6]], since the GPCMV crosses the placenta, causing infection and disease *in utero*. One significant limitation of the GPCMV model is the resistance of GPCMV to ganciclovir, the most clinically useful of the therapeutic agents employed for management of human CMV infections [7,8]. GPCMV is susceptible, however, to cidofovir, and its cyclic cogener, cHPMPC [8]. Treatment of guinea pigs with cyclic cidofovir has been shown to be safe and effective in ameliorating GPCMV disease, including labyrinthitis and associated SNHL [9,10]. However, to date there is no published information about its efficacy in prevention of congenital GPCMV infection in pregnant guinea pigs. These studies were therefore undertaken to evaluate the potential efficacy of cyclic cidofovir against congenital GPCMV infection, using an enhanced green fluorescent protein (eGFP)-tagged virus [11], to aid in the detection of vertically transmitted infection in pups born to animals challenged with GPCMV in the third trimester of pregnancy.

## Results

### Characterization of vAM403: genome structure and virion polypeptides

The details of construction of the eGFP-tagged recombinant GPCMV used in this study, vAM403, have been previously described [11]. Briefly, this virus was generated using *gpt*-mediated mutagenesis, with insertion of an eGFP/*gpt *cassette in the *Hin*d III locus of the GPCMV genome, followed by clonal purification of recombinant stock. Previous work indicated that this virus replicated with wild-type kinetics *in vitro*, and was capable of widespread dissemination and attendant mortality in cyclophosphamide-immunocompromised guinea pigs *in vivo *[12].

**Figure 1:**
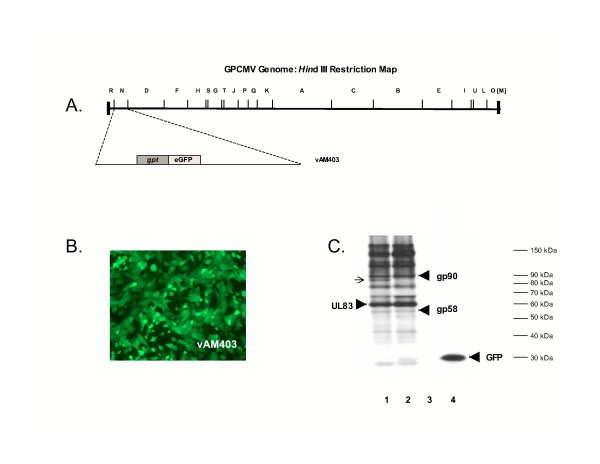
**Characterization of recombinant, eGFP-expressing guinea pig cytomegalovirus (GPCMV) used for *in vivo *antiviral studies**. An eGFP-expressing, recombinant GPCMV was generated using *gpt *selection to enable detection of infection in the guinea pig model of congenital CMV infection [11]. Panel A, genome map of GPCMV genome (*Hin*d III digestion profile) indicating site of insertion of eGFP/*gpt *cassette. Insertion of this cassette into GPCMV genome did not disrupt any predicted conserved CMV open reading frames, based on homology comparisons with other CMVs and BLAST search [6]. Panel B, fluorescence microscopy analysis of vAM403 virus in tissue culture. GPL cells were inoculated with recombinant virus and visualized by FITC filter 96 hours post-infection. Panel C, analysis of vAM403 protein expression and comparison to wild-type GPCMV. GPL cells infected with either wild-type virus (lanes 1, 3) or vAM403 (lanes 2, 4) were labelled with ^35^S-methionine/cysteine, and immunoprecipitated with either polyclonal anti-GPCMV antisera (lanes 1, 2) or a monoclonal antibody against eGFP (lanes 3, 4). Position of molecular weight markers is indicated. Precipitation with serum from a GPCMV seronegative guinea pig did not precipitate any viral proteins (data not shown). The pattern of proteins recognized by immune serum against wild-type and vAM403 virus is virtually identical, with exception of a band of ~85 kDa present in wild-type infected cells, but not present in vAM403-infected cells (arrow). Known positions of migration of gB complex (gp90 and gp58) are indicated, as is the position of migration of UL83 homolog (arrowheads). The eGFP monoclonal antibody precipitated the ~30 kDa eGFP protein from vAM403 cells (lane 4, arrowhead), but not wild-type cells (lane 3).

The structure of this recombinant virus is summarized in Fig. 1a. Although this virus was known to produce disease *in vivo*, virus-associated proteins encoded by this virus had not been previously characterized. The insertion in the *Hin*d III locus was not anticipated to result in disruption of any known proteins encoding by other betaherpesviruses, based on sequence analysis and comparison of this sequence to the BLAST database [6]. To experimentally characterize the protein profile of the vAM403 virus, radio-immunoprecipitation assays were performed with polyclonal anti-GPCMV antibodies, using polypeptides purified from ^35^S-labeled cells. Guinea pig lung (GPL) cells were infected either with wild-type virus, or the vAM403 recombinant. These studies indicated that the polypeptide profile of the vAM403 virus, assessed by immunoprecipitation assay, was essentially identical to that of wild-type virus, with the exception of high-level expression of the eGFP protein, which was confirmed using an eGFP-specific monoclonal antibody (Fig. 1c). In addition, in some experiments, vAM403-inoculated cells had reduced levels of expression of a protein of ~85 kDa (Fig. 1c, arrow). Expression of well-characterized GPCMV proteins, including the glycoprotein B and UL83 (pp65) homologs, was unchanged in the vAM403 mutant, compared to wild-type virus (Fig. 1c). These studies, coupled with previous vAM403 genome structure characterization by gel electrophoresis and Southern blot [11] as well as biological characterization *in vivo *[12], suggested that insertion of the eGFP/*gpt *cassette had no major influence on the biology of the virus, and no significant impact on the expression of immunogenic, virus-associated polypeptides.

### Maternal and pup outcomes following vAM403 challenge in pregnancy

The vAM403 virus had previously been shown to induce disseminated GPCMV disease, and mortality, in cyclophosphamide-immunosuppressed guinea pigs [12]. To assess the virulence of this virus in pregnant guinea pigs, outbred Hartley strain guinea pigs (n = 4) were challenged with 1 × 10^7 ^pfu of vAM403 subcutaneously in the early 3^rd ^trimester of pregnancy and pregnancy outcomes monitored. In this initial study of GPCMV-challenged dams, vertical transmission of virus was noted in 50% of pups, as assessed by viral culture of pup tissue homogenates (data not shown). In addition, placentas retrieved from two pregnant dams revealed evidence of GPCMV by viral co-culture and fluorescent microscopy (data not shown). Thus, these observations reinforced the observations that vAM403 could be virulent in immunosuppressed non-pregnant animals, and provided a framework for antiviral intervention studies with cyclic cidofovir.

### Cyclic cidofovir reduces mortality and prevents GPCMV transmission

To assess the ability of cyclic cidofovir therapy to interrupt vertical GPCMV transmission, the eGFP-tagged vAM403 virus was used to challenge pregnant animals in the early 3^rd ^trimester. A total of 9 dams were challenged with vAM403 virus (dose, 1 × 10^6 ^pfu, administered subcutaneously) and had evaluable pregnancy outcomes. A lower dose of GPCMV was used in this study than in the pilot study, in order to minimize the risk of maternal mortality prior to delivery, so that the impact of antiviral therapy on congenital infection could be clearly discerned. Cyclic cidofovir (20 mg/kg) was administered to 4 animals, and saline diluent (negative control) was administered to 5 animals. Antiviral drug was administered in a single dose, via intraperitoneal route, 24 hours after subcutaneous viral challenge with vAM403 virus. Animals were challenged in the early-third trimester, approximately 3 weeks prior to anticipated delivery. Following viral challenge and antiviral therapy administration, pregnancies were monitored and pregnancy outcomes (pup and maternal mortality) were assessed. In addition, congenital infection rates were determined by co-cultivation of lung, liver and spleen from pups, on GPL cells, and GPCMV was detected by monitoring of cells by fluorescence microscopy, with examination for eGFP-positive CMV plaques (Fig. 1B).

The results of these analyses are summarized in Tables 1 and 2. The overall magnitude of combined GPCMV-induced maternal and fetal mortality in the placebo-treated, GPCMV-infected group was 5/25 animals (20%; includes 4 pups, and 1 dam which died shortly after delivery). In contrast, in animals treated with cyclic cidofovir, the mortality was 0/21 dams and pups (0%; p = 0.05 vs. placebo group, Fisher's exact test). Two of five litters in the control group had pup mortality (40%), in contrast to no pup mortality in any of four litters in the cyclic cidofovir-treated animals. In control pups, 19 pups were available for culture, and in cyclic cidofovir-treated animals, 16 pups were available for culture. Of these, 5 pups in the control group (26%) were culture-positive for GPCMV, compared to 0/16 pups (all liveborn) from the cyclic cidofovir-treated group (p < 0.05, Fisher's exact test). Among maternal cultures, 2/5 (40%) of control dams were culture-positive from any visceral organ, compared to 0/4 (0%) of cyclic cidofovir-treated dams. Overall, cyclic cidofovir eliminated recovery of GPCMV by viral culture from visceral organ in both dams and pups (7/24 of control dams and/or pups were culture positive, versus 0/20 animals in cyclic cidofovir group; p = 0.01, Table 2). Thus, antiviral therapy of the pregnant dam with cyclic cidofovir completely eliminated congenital transmission of GPCMV to the fetus, and also prevented visceral dissemination to maternal tissues, as assessed by viral culture techniques.

**Table 1:** Impact of Cyclic Cidofovir On GPCMV-Associated Mortality Following GPCMV (vAM403) Challenge During Third Trimester of Pregnancy. Animals challenged in the early 3^rd ^trimester of pregnancy were monitored for outcome by assessment of pup mortality rates (liveborn versus dead pups) and GPCMV-associate maternal mortality. In 5 litters in the placebo (saline) group, there were 2 litters with dead pups (40%) and the overall mortality was 20%. One dam also died, shortly after childbirth, and had disseminated GPCMV infection (liver, lungs, spleen). In contrast, cyclic cidofovir-treated animals had no pup or maternal mortality.

**Control (Saline)**	**Live**	**Dead**	**%**
Maternal Mortality	4	1	20%
Pup Mortality	16	4	20%
Total Mortality	20	5	20%
**Cyclic Cidofovir**	**Live**	**Dead**	**%**
Maternal Mortality	4	0	0
Pup Mortality	21	0	0
Total Mortality	25	0	0 *

* p = 0.05 vs. placebo, Fisher 's Exact Test

**Table 2:** Impact of Cyclic Cidofovir On Maternal and Congenital GPCMV Infection Rates. To assess the extent of maternal and congenital GPCMV dissemination, both maternal and pup organs (liver, spleen, lung) were collected, both from animals who died during course of experiment, and following euthanasia within 72 hours of delivery, and homogenates were co-cultured on GPL cells. Congenital transmission was defined by any positive pup culture from any organ. The rate of congenital GPCMV transmission was 26% in placebo-treated controls, versus 0% in cyclic-cidofovir treated dams. Among maternal cultures, 2/5 (40%) of control dams were culture-positive, compared to 0/4 (0%) of cidofovir-treated dams. GPCMV was isolated from 3 dead pups, and 2 live pups, from 3 different litters in the control group, including both dams which were culture-positive from visceral organs, and, interestingly, one control dam which was culture-negative from visceral organs.

Group	Culture Positive Dams	Culture Positive Pups	Total Culture Positive Animals	Congenital Transmission Rate
Placebo	2/5	5/19	7/27	26%
Cyclic Cidofovir	0/4	0/16	0/20^§^	0%*

* p < 0.05 vs. placebo, Fisher 's Exact Test

^§^p = 0.01 vs. placebo, Fisher 's Exact Test

## Discussion

Interventions are urgently needed for the problem of congenital CMV infection. Although CMV vaccines are in various stages of preclinical development, none are currently licensed for human use. Antiviral agents for CMV, in contrast, are available, and have already been shown to be useful in the management of congenital and perinatal CMV infections [2,13]. However, there is no conclusive evidence that antiviral treatment of pregnant women is capable of interrupting CMV transmission. Since the guinea-pig model of CMV infection is the most authentic non-primate animal model for study of interventions against congenital CMV infection [14], these studies were undertaken to test the hypothesis that an effective antiviral agent might be capable of reducing transmission of CMV during pregnancy.

The intrinsic resistance of GPCMV to the most commonly used CMV antiviral, ganciclovir, precludes evaluation of this agent for efficacy against congenital GPCMV infection [7,8]. However, the cyclic cogener of cidofovir is highly active against GPCMV *in vitro *and, therapeutic against disseminated GPCVM disease, and against viral labyrinthitis and SNHL, *in vivo *[8,9]. The molecular basis for the improved activity of this agent against GPCMV, compared to ganciclovir, is not clear, but it may derive from the fact that cidofovir does not require phosphorylation by the CMV U_L_97 kinase for antiviral activity, in contrast to ganciclovir. Since the GPCMV U_L_97 homolog has significant divergence (compared to HCMV) of amino acid sequence in catalytic domains of the protein important for ganciclovir phosphorylation, this may provide a molecular explanation for the decreased susceptibility of GPCMV to this nucleoside antiviral [15]. In any case, cyclic cidofovir has been found to be not only highly effective against GPCMV, but also non-toxic to animals [8]. The study described in this report found that, following challenge with an eGFP-tagged strain of GPCMV (vAM403), guinea pigs that received cyclic cidofovir therapy (20 mg/kg, via intraperitoneal route) had reduced overall maternal and pup mortality, compared to placebo-treated controls. This difference in overall mortality was statistically significant, with a reduction from 5/25 (20%) in the placebo group, to 0/21 (0%) animals in the cyclic cidofovir-treated group (p = 0.05, Fisher's exact test). Furthermore, these studies found that antiviral therapy resulted in elimination of congenital GPCMV transmission. In untreated controls, congenital GPCMV infection was demonstrated in 5/19 pups (26%), versus 0/16 pups in the cyclic cidofovir group (p < 0.05, Fisher's exact test). These are the first data to demonstrate that antiviral therapy during pregnancy can prevent congenital CMV transmission. These observations provide support for more detailed examination of cyclic cidofovir, and other antiviral agents, in this small animal model of CMV transmission. These data may also provide indirect support for potential clinical trials of antiviral interventions in pregnant women at high risk of transmission of CMV to the fetus, although caution should be exercised in human studies, given the potential for toxicity of these agents when used during pregnancy [13]. A recent study of passive immunization of women at high risk for giving birth to infants with symptomatic congenital CMV infection suggested that antibody therapy *in utero *resulted in improved pregnancy outcomes [16]. Future controlled clinical trials will be required to further assess the value as well as the potential toxicities of antiviral interventions targeting CMV transmission in the pregnant patient.

An important observation from these studies was the demonstration of the utility of using an eGFP-tagged, recombinant CMV in the experimental modelling of congenital CMV infection. Previous studies using an eGFP-tagged rhesus macaque CMV have demonstrated that the resultant recombinant virus retained full pathogenicity in the experimentally inoculated fetus *in utero*, but this required direct inoculation of the fetus [17]. It was therefore of considerable interest to determine if the recombinant GPCMV used in these studies was capable of resulting in transplacental infection and conferring maternal and fetal disease in pregnant guinea pigs without direct fetal inoculation. Characterization of the eGFP-tagged GPCMV recombinant, vAM403, indicated that insertion of the eGFP/*gpt *cassette into the GPCMV genome, in the nonessential *Hin*d III "N" locus, had minimal impact on viral protein expression compared to wild-type virus, as assessed by SDS-PAGE. This observation, coupled with the apparent absence of any conserved betaherpesvirus proteins encoded by this region of the genome, as ascertained by DNA sequence analyses [6], suggested that this virus would have the full potential for virulence in animals. Consistent with this prediction, this virus was found to be fully capable of transplacental transmission, with attendant maternal and pup mortality, following subcutaneous inoculation of pregnant guinea pigs. The future use of other eGFP-tagged viruses should facilitate study of congenital GPCMV infection in this model. Targeted mutagenesis of the GPCMV genome cloned in *E. coli *as an infectious bacterial artificial chromosome (BAC) [18] will facilitate future, more detailed evaluation of the role of viral genes in pathogenesis of maternal-fetal infection.

## Conclusion

Targeted insertion of an eGFP/*gpt *cassette into the *Hin*d III 'N' region of the GPCMV genome, using homologous recombination techniques, resulted in generation of a virus which retained full pathogenicity in pregnant guinea pigs, including the potential for transplacental transmission. The recombinant virus, vAM403, retained a protein synthesis profile similar to wild-type virus. The expression of the eGFP reporter cassette in infected cells resulted in the efficient and rapid detection of virus in experimentally inoculated animals, including congenitally infected, newborn pups. Recombinant virus was capable of infecting the fetus, inducing disease and mortality, following experimental inoculation of pregnant guinea pigs. Maternal and fetal GPCMV disease and mortality was abrogated by antiviral therapy with cyclic cidofovir. In addition, cyclic cidofovir therapy eliminated congenital transmission of GPCMV. These are the first data confirming that antiviral treatment administered during pregnancy is capable of modifying the course of CMV disease and preventing vertical transmission of CMV in a relevant animal model of congenital infection, and provides support for future clinical trials of antivirals in pregnant patients.

## Methods

### Analysis of eGFP/*gpt *recombinant virus

The construction of the vAM403 recombinant virus is reviewed in detail elsewhere [11]. Recombinant, eGFP-expressing virus was plaque-purified by limiting dilution, and a workpool of viral stock was generated by cultivation in guinea pig lung (GPL) cells (ATCC CCL158) and maintained in F-12 medium supplemented with 10% fetal calf serum (Gibco-BRL). Viral stocks were prepared for *in vivo *studies, and DNA was extracted from the cellular pellet by sodium iodide ultracentrifugation and analyzed by agarose gel electrophoresis and Southern blot analysis as previously described [11], to confirm the genome structure of the eGFP/*gpt *cassette (data not shown). To examine polypeptides synthesized by wild-type and vAM403 recombinant GPCMV, GPL monolayers were inoculated at a multiplicity of infection of 10 plaque-forming units/cell and subsequently were pulse-labelled for 4 hours with ^35^S-methionine/cysteine (Express^® ^protein labelling mix, New England Nuclear), at a concentration of 50 μCi/ml tissue culture medium, at 96 hours post-infection. Cellular lysates were prepared and subjected to SDS-PAGE, or to RIP-PAGE using either a polyclonal anti-GPCMV antibody [12] or a monoclonal anti-eGFP antibody (Clontech), along with *Staphylococcus aureus *protein A/sepharose, to pellet protein-antibody complexes. Following separation of proteins, autoradiography of SDS-PAGE gels was carried out, in order to analyze protein expression patterns in wild-type and vAM403- infected cells.

### Viral stock preparation and animal challenge experiments

For *in vivo *work, a clonal stock of vAM403 virus was prepared following four rounds of limiting dilution cloning. Virus was prepared from tissue culture supernatants and titered using standard plaque reduction assay and fluorescence microscopy to enumerate plaques. For animal experiments, pregnant outbred Hartley guinea pigs were purchased from Harlan Laboratories (Indianapolis, IN) and confirmed to be GPCMV-seronegative by ELISA assay. Guinea pigs were housed in an AALAC-accredited vivarium, and all animal experiments were approved by the institutional animal use committee. Animals were challenged with GPCMV (10^6 ^PFU of vAM403 viral stock, administered subcutaneously) in the early third trimester of pregnancy, approximately 21 days prior to anticipated delivery, and were monitored daily thereafter (including daily weights, assessment of food intake, and monitoring for signs of illness including listlessness, hair loss, and decreased activity. Pregnancy outcomes were monitored, including days to delivery, liveborn and stillborn pups, and pup weights. For antiviral studies, dams received either cyclic cidofovir, 20 mg/kg in saline diluent (10 mg/ml concentration), or an equivalent volume of saline placebo. Cyclic cidofovir (a gift from Gilead Pharmaceuticals) was administered intraperitoneally, in a single dose, 24 hours after viral inoculation.

To monitor for congenital GPCMV infection, all stillborn pups, and deceased dams, were dissected for harvest of spleen, liver, and lung, with subsequent co-culture of homogenates (10% w/v) on GPL monolayers. In addition, most liveborn pups, and all surviving dams, were humanely euthanized within 72 hours of delivery, and liver, spleen and lung similarly harvested for viral culture. Cultures were considered positive if they showed characteristic CMV plaques and exhibited fluorescence. Plates were held for 21 days, but most positive cultures exhibited fluorescence within 72 hours of inoculation. No eGFP-negative viral plaques were observed, suggesting that the passage of vAM403 in vivo did not result in deletion of the eGFP/*gpt *cassette. Study outcomes were compared by Fisher's exact test, using the InStat^® ^statistical analysis program (GraphPad software).

## Competing interests

The author(s) declare that they have no competing interests.

## Authors' contributions

All authors participated in the study design, carrying out the experiments, data analysis, and manuscript preparation.
